# AAV-vector based gene therapy for mitochondrial disease: progress and future perspectives

**DOI:** 10.1186/s13023-022-02324-7

**Published:** 2022-06-06

**Authors:** Allison R. Hanaford, Yoon-Jae Cho, Hiroyuki Nakai

**Affiliations:** 1 Center for Integrative Brain Research, Seattle Children’s Reserach Institute, Seattle, WA 98101 USA; 2 Papé Family Pediatric Research Institute, Oregon Health and Science University, Portland, OR 97239 USA; 3 Division of Pediatric Neurology, Doernbecher Children’s Hospital, Oregon Health and Science University, 3181 SW Sam Jackson Park Rd, Portland, OR 97239 USA; 4 Department of Molecular and Medical Genetics, Oregon Health and Science University, Portland, OR 97239 USA; 5 Department of Molecular Immunology and Microbiology, Oregon Health and Science University, Portland, OR 97239 USA; 6 Division of Neuroscience, Oregon National Primate Research Center, Beaverton, OR 97006 USA; 7 Knight Cancer Institute, Oregon Health & Science University, Portland, OR 97239 USA

**Keywords:** Gene therapy, Mitochondrial disease, AAV

## Abstract

Mitochondrial diseases are a group of rare, heterogeneous diseases caused by gene mutations in both nuclear and mitochondrial genomes that result in defects in mitochondrial function. They are responsible for significant morbidity and mortality as they affect multiple organ systems and particularly those with high energy-utilizing tissues, such as the nervous system, skeletal muscle, and cardiac muscle. Virtually no effective treatments exist for these patients, despite the urgent need. As the majority of these conditions are monogenic and caused by mutations in nuclear genes, gene replacement is a highly attractive therapeutic strategy. Adeno-associated virus (AAV) is a well-characterized gene replacement vector, and its safety profile and ability to transduce quiescent cells nominates it as a potential gene therapy vehicle for several mitochondrial diseases. Indeed, AAV vector-based gene replacement is currently being explored in clinical trials for one mitochondrial disease (Leber hereditary optic neuropathy) and preclinical studies have been published investigating this strategy in other mitochondrial diseases. This review summarizes the preclinical findings of AAV vector-based gene replacement therapy for mitochondrial diseases including Leigh syndrome, Barth syndrome, ethylmalonic encephalopathy, and others.

## Introduction

### Basic genetics and pathobiology of mitochondrial diseases

Mitochondrial diseases are a heterogeneous group of disorders resulting from defects in mitochondrial function. Mitochondria are double-membrane-bound organelles that generate cellular energy in the form of ATP by oxidative phosphorylation via the four complexes of the electron transport chain (ETC) and ATP synthase. Mitochondria also play critical roles in apoptosis, metabolic regulation, and biosynthesis of macromolecules [1]. Mitochondria possess their own genome that encodes 13 proteins involved in the oxidative phosphorylation system, 22 transfer RNAs (tRNAs) and 2 ribosomal RNAs (rRNAs) [2]. All the genes in the mitochondrial genome are transcribed and translated within the mitochondria. The remainder of the 1000 or so genes involved in mitochondrial functions are coded in the nuclear genome. As of 2020, pathogenic mutations have been identified in 36 of the 37 mitochondrial DNA (mtDNA)-encoded genes and in 295 nuclear genes [2]. Mitochondrial disease can be caused by mutations in genes coding for mitochondrial proteins, rRNAs, or tRNAs. Inheritance of mitochondrial diseases can be maternal, autosomal recessive, autosomal dominant, X-linked or sporadic.

Mitochondrial diseases most commonly affect the tissues that require high levels of cellular ATP production for proper function, such as the nervous system, heart, skeletal muscle, and liver [1]. Thus, mitochondrial diseases tend to be multi-system disorders and classified as syndromes diagnosed based on symptomatology. This presents challenges due to the extreme variation in symptoms and genetics. On one hand, different mutations in the same gene can cause very different phenotypes. For example, certain mutations in the *Apoptosis-Inducing Factor Mitochondrial Associated 1* (*AIFM1)* gene lead to severe encephalomyopathy and death in infancy [3], while other mutations only cause deafness [4]. On the other hand, Leigh syndrome can be caused by mutations in 75 different genes [5].

Mitochondrial diseases are also very rare, with an incidence of around 12.5 per 100,000 in adults [6] and about 4.7 per 100,000 in children [7]. Treatment is limited to supportive care for the majority of mitochondrial diseases. Issues like cardiomyopathy and seizures can be managed with standard medications. Many patients also receive a variety of physical, occupational, and speech therapies to improve their function and quality of life. Mitochondrial replacement therapy (MRT) has been proposed as a preventative therapy for mitochondrial diseases caused by mitochondrial DNA mutations [8]. In MRT, the pronuclei created from the oocyte and sperm of parents with a history of mitochondrial disease is removed and placed into a pronuclei-depleted donor oocyte [8]. MRT has resulted in live human births, but it remains controversial and has not yet been approved in the United States [8]. A variety of other therapies are being investigated for mitochondrial diseases, including new antioxidants, drugs to activate mitochondrial biogenesis and regulate mitophagy, enzyme replacement therapy and gene therapy [9].

### AAV vector-based gene therapy

One of the most popular vectors for gene replacement therapy is derived from adeno-associated virus (AAV). AAV is a small, non-pathogenic virus that consists of a capsid and a single-stranded DNA genome. The wild-type AAV genome is about 4.7 kilobases (kb) in size and consists of the *rep* and *cap* genes flanked by inverted terminal repeats (ITRs). For therapeutic purposes, the entire AAV genome, except for the ITRs, can be replaced by a therapeutic genetic payload to make recombinant AAV vectors for gene delivery that are devoid of the majority of the genetic components in the viral genome [10]. Their relatively small size means AAVs have a limited packaging capacity of about 4.7 kb. The promoter, regulatory elements and transgene must all be less than 4.7 kb in size, limiting the size of the transgene that can be delivered in a single AAV capsid. Delivery of large transgenes by AAV requires alternative strategies [11].

Recombinant AAV vectors normally are not equipped with integration machinery, and thus vector genomes rarely integrate into the host genome, reducing the risk of insertional mutagenesis [12]. Long term follow-up of a small cohort of dogs treated with an AAV-vector based therapy for hemophilia A found clonal expansion of integrated AAV vectors 10 years post treatment [13]. Though no treated animal had any adverse effects or malignancies, this study indicates the importance of long-term monitoring in AAV treatment studies [13].

When AAV vectors transduce a cell, the AAV vector particles are trafficked to the nucleus, where single-stranded vector genomes are released from the capsid and converted to double-stranded DNA. The double-stranded vector genome can be transcribed and translated [10] and persists long-term as extrachromosomal DNA in the nuclei, as long as cells do not divide [12, 14, 15]. This results in long-term expression of therapeutic molecules from AAV vectors in quiescent tissues, the major target for gene therapy for a variety of human diseases including mitochondrial diseases. Importantly, AAV vectors have the ability to transduce various tissues efficiently when the vector is injected directly into the body. AAV vectors have become increasingly popular as the most efficient gene delivery vectors for in vivo gene therapies.

Thirteen naturally occurring AAV capsid serotypes found in humans and non-human primates have been characterized [16]. These serotypes show diverse cell and tissue tropism. The tissue and cell tropism are important considerations when developing an AAV vector-based gene therapy. Of the most commonly discussed serotypes in this review, AAV9 can cross the blood–brain barrier and transduce a variety of cell types found in the central nervous system (CNS) [17], AAV8 shows high tropism for hepatocytes [18], and AAV2 tropism is broad. An active area of research is engineering novel capsids with desirable properties, such as specificity for a specific cell or tissue type. Various engineering strategies have led to identification of novel capsids with high-level retinal transduction [19] and high-level brain transduction [20].

There are currently two FDA-approved AAV vector-based gene therapy products: Luxturna (an AAV2-based product for Leber congenital amaurosis type 2, a degenerative retinal disease caused by mutations in the *RPE65* gene) and Zolgensma (an AAV9-based product for spinal muscular atrophy type 1, a severe neuromuscular disease caused by mutations in the *SMN1* gene). More gene therapy products will likely be approved in the coming years.

Almost 90% of mitochondrial diseases are caused by a mutation in a single nuclear gene. As monogenic diseases caused by nuclear gene defects are the most amenable to AAV vector-mediated gene replacement, mitochondrial diseases are good candidates for this therapeutic modality*.* This review will discuss the preclinical research on AAV vector-mediated gene replacement therapy in mitochondrial diseases, and its potential for treating these devastating conditions. The review is organized by disease, and each section includes a brief description of disease pathology and current therapeutic options prior to discussing relevant AAV vector-mediated gene therapy studies. The investigation of AAV vector-based gene therapy in mitochondrial disease has been conducted almost exclusively in mouse models, as opposed to cell-based models such as iPSCs or organoids. The review will focus on mitochondrial diseases where AAV-based gene therapies have been pre-clinically tested in mice: Barth syndrome, Friedreich ataxia, *NDUFS4*, *NDUFS3*, and *SURF1*-related Leigh syndrome, ethylmalonic encephalomyopathy, three mitochondrial DNA depletion disorders (mitochondrial neurogastrointestinal encephalomyopathy (MNGIE), *MPV17* deficiency, and *TK2* deficiency), Leber hereditary optic neuropathy and S*LC25A46*-related neuropathy.

## Pre-clinical studies on AAV vector-based gene replacement therapy in mitochondrial diseases

Please note that AAV vectors are named by the following convention in this review: capsid type-promoter-gene (*e.g.*, AAV9-CAG-hTAZ). Table 1 summarizes all the preclinical studies discussed.

**Table 1:** Summary of the preclinical studies discussed in this review

Disease	Pre-clinical studies	AAV capsid serotype	Dose	Age/route*	Results
Barth syndrome	Suzuki-Hatano et al*.* (2019). *Hum Gene Ther* [23]	AAV9	1 × 10^13^ vg/kg	P1(IV) 3 months (IV)	Symptom improvement
	Wang et al*.* (2020). *Circ Res* [25]	AAV9	1 × 10^13^ vg/kg 2 × 10^13^ vg/kg	P1 (SubQ) 20 days (RO) 2 months (RO)	Neonatal administration rescued neonatal death, prevented development of cardiac phenotype. Adult administration prevented or reversed cardiac dysfunction
Friedreich ataxia	Perdomini et al*.* (2014). *Nat Med *[36]	AAVrh10**	5.4 × 10^13^ vg/kg	3 weeks (IV)	Pre-symptomatic administration prevents phenotype development. Post-symptomatic treatment reversed disease
	Piguet et al*.* (2018). *Mol Ther* [39]	AAV9	5 × 10^13^ vg/kg 1 × 10^10^ vg × 3 (brain)	3.5 weeks (IV) 7.5 weeks (IV/IC)	IV only partially improved phenotype. IV and IC treatment resulted in symptom improvement
	Gerard et al*.* (2014). *Mol Ther Methods Clin Dev* [41]	AAV9	6 × 10^9^ to 6 × 10^11^ vg	5–9 days (IP)	Increased survival and reduced symptoms
Leigh syndrome	Di Meo et al*.* (2017). *Gene Ther* [49]	AAV9	1 × 10^12^, 2 × 10^12^ vg 1.5 × 10^11^, 3 × 10^11^ vg	P1 (IV/ICV)	The combination of IV and ICV treatment improved survival and reduced symptoms. IV or ICV alone did not increase survival or improve symptoms
	Reynaud-Dulaurier et al*.* (2020). *Brain* [50]	PHP.B	1 × 10^12^ vg	1 month (IV)	Increased survival and delayed disease progression
	Silva-Pinheiro et al*.* (2020). *Mol Ther Methods Clin Dev* [51]	PHP.B	1 × 10^12^vg, 2 × 10^12^ vg	26 or 28 days (IV)	Increased survival and delayed disease progression
	Pereira et al. (2020). *EMBO Mol. Med* [54]	AAV9	1.25 × 10^15^ vg/kg (juveniles) 1.66 × 10^15^ vg/kg (adults)	15–8 days (RO) 2 months (RO)	Treatment of juveniles prevents development of disease. Treatment of adults corrected established disease
	Ling et al. (2021) *Mol Ther* [58]	scAAV9	8 × 10^11^ vg, 2 × 10^11^ vg, 8x^11^vg + 8x^11^vg (IV + IT)	4 weeks (IT or IV + IT)	Increased CIV activity in brain, muscle, and liver Decreased blood lactate following exhaustive exercise
Ethylmalonic encephalopathy	Di Meo et al*.* (2012). *EMBO Mol Med*.[71]	AAV8	4 × 10^13^ vg/kg	21 days (IV)	Increased survival and improved motor function
MNGIE	Torres-Torronteras et al*.* (2018). *Hum Gene Ther*.[85]	AAV8	2 × 10^11^, 1 × 10^12^, 2 × 10^12^ vg/kg	8–12 weeks (IV)	Sustained lowering of plasma dThd and dUrd levels
	Cabrera-Perez et al*.* (2019). *Hum Gene Ther*. [86]	AAV8	5 × 10^10^, 2 × 10^11^, 5 × 10^11^ vg/kg (TBG) 5 × 10^10^, 2 × 10^11^, 5 × 10^11^, 10^12^, 2 × 10^12^ vg/kg (AAT) 2 × 10^11^, 5 × 10^11^, 1 × 10^12^, 2 × 10^12^ vg/kg (PGK, HLP)	8–12 weeks (IV)	Sustained lowering of plasma dThd levels. Liver-specific promoters resulted in the longest supression of dThd levels
	Vila-Julia et al. (2020). EBioMed [88]	AAV8	5 × 10^11^, 1 × 10^12^, 2 × 10^12^, 1 × 10^13^ vg/kg (TBG) 2 × 10^12^, 1 × 10^13^ vg/kg (AAT, HLP)	8–11 weeks (IV)	Sustained lowering of plasma dThd and dUrd levels, modestly improved motor performance, and modestly decreased ventricular volume
MPV17	Bottani et al*.* (2014). *Mol Ther*.[90]	AAV8	4 × 10^12^, 4 × 10^13^ vg/kg	2 months (IV)	Treatment after beginning ketogenic diet improved liver phenotype. Treatment prior to ketogenic diet prevented further liver damage
TK2 deficiency	Lopez-Gomez et al. (2021). *Ann Neurol* [101]	AAV9, AAV2	2.1 × 1011, 4.2 × 1011	P1 (RO)	Improved survival and motor function
SLC25a46	Yang et al*.* (2020). *Hum Mol Gen *[108]	AAV-PHP.eB	1 × 10^11^, 2 × 10^14^ vg/kg	P2 (IV)	Improved survival, development of less severe phenotype
Leber hereditary optic neuropathy	Yu et al*.* (2012). *Proc Natl Acad Sci USA* [119]	MTS-AAV2	1 × 10^8^ vg (intravitreal)	N/A***	Pre-treatment protected against vision loss
	Yu et al*.* (2018). *Sci Rep*.[122]	MTS-AAV2	4.4 × 10^8^ vg	3 months (intravitreal)	Stably improved visual function

*IV, intravenous; SubQ, subcutaneous; RO, retro-orbital; IC, intracerebral; ICV, intracerebral ventricular; IT, intrathecal

**Voyager Therapeutics has an AAVrh10-based therapy for FRDA (VY-FXN01) in preclinical development

***The phenotype in this study is induced by injection of AAV2-R340H-hMT-ND4, which can be done at any time. Timing of treatment is important as it relates to injection of mutant ND4

### Barth syndrome

**Fig. 1:**
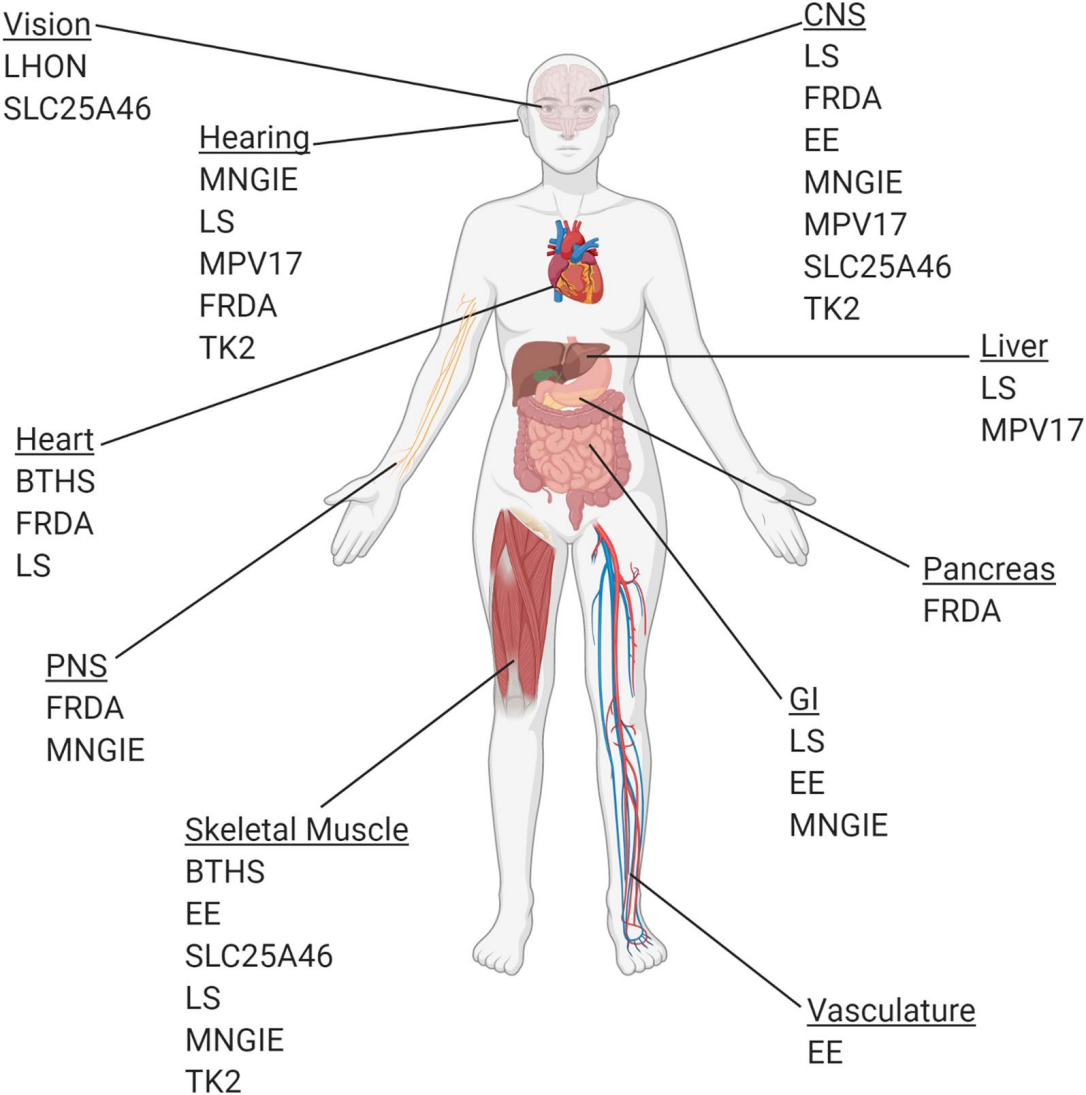
Organ systems affected by the mitochondrial diseases discussed in this review. BTHS, Barth syndrome; FRDA, Friedreich ataxia; LS, Leigh syndrome; EE, ethylmalonic encephalomyopathy; MNGIE, mitochondrial neurogastrointestinal encephalopathy; LHON, Leber hereditary optic neuropathy; TK2, thymidine kinase 2 deficiency. Created with BioRender.com

**Fig. 2:**
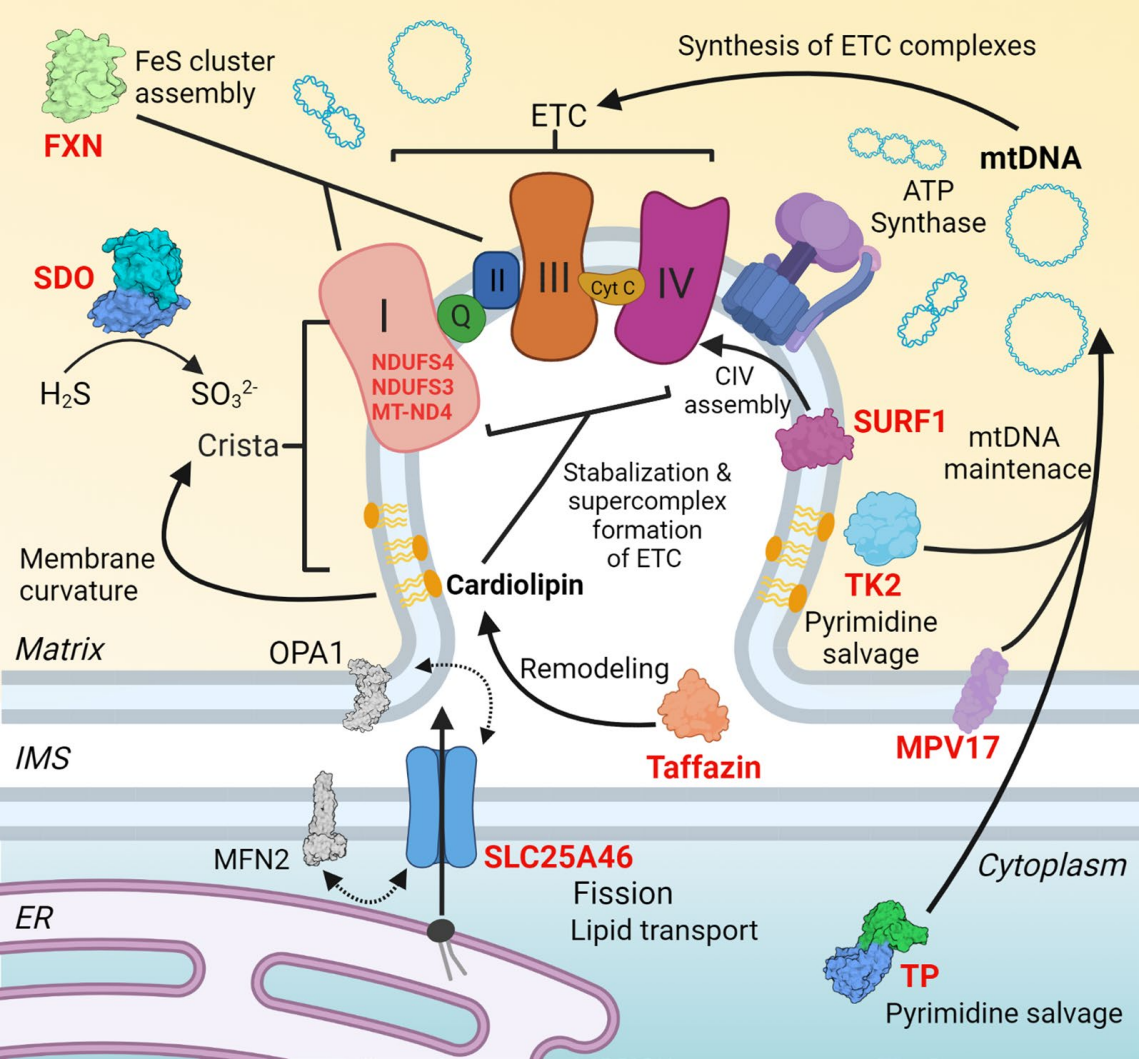
Close up of a crista of a mitochondria showing the processes affected by the discussed mitochondrial diseases. Red text indicates mutations have been found in the particular gene that can cause mitochondrial disease. FXN, Frataxin, mutations result in Friedrich ataxia; SDO, sulfur dioxygenase, mutations result in ethylmalonic encephalomyopathy; TP, thymidine phosphorylase, mutations result in mitochondrial neurogastrointestinal encephalopathy (MNGIE); TK2, thymidine kinase, mutations result in TK2 deficiency; Taffazin mutations result in Barth syndrome; NDUFS4, NDUFS3, and SURF1, mutations can result in Leigh syndrome; MT-ND4, NADH dehydrogenase subunit 4, mutations result in Leber hereditary optic neuropathy (LHON); MPV17, mutations can cause a mtDNA depletion syndrome; SLC25A46, mutations can result in mitochondrial disease. IMS, intermembrane space. ER, endoplasmic reticulum. Protein structures for FXN, SDO, MFN2, OPA1, Taffazin, and TP are from the RCSB Protein Databank (rcsb.org)  [149]. Created with BioRender.com

Barth syndrome (BTHS) is caused by mutations in *TAZ*, which results in cardiomyopathy, neutropenia, and skeletal muscle weakness (Fig. 1) [21]. Patients rarely survive past their forties, and death is usually due to cardiomyopathy [21]. The Taffazin protein is an acyltransferase located in the inner mitochondrial membrane responsible for remodeling monolysocardiolipin (MLCL) to its mature form of cardiolipin (CL) (Fig. 2) [22]. The unique structure of cardiolipin enables the curvature of the inner mitochondrial membrane to form cristae and stabilizes the individual complexes of the ETC, facilitating oxidative phosphorylation [22]. BTHS mitochondria have an increased MLCL/CL ratio that causes instability of the inner mitochondrial membrane, ultimately resulting in a decreased efficiency in ATP production [22].

Inducible *Taz-*knockdown mice treated with a cardiotropic AAV serotype (AAV9, which also transduces the liver, skeletal muscle, brain, and other tissues) encoding human *TAZ* under control of a muscle-specific promoter (*desmin)* showed improved activity levels, muscle strength, cardiac function and mitochondrial structure [23]. The hearts of treated animals had a protein expression profile similar to wild-type animals, indicating that AAV vector-based *TAZ* gene replacement therapy corrected the underlying protein deficiencies caused by *Taz* knockdown [24]. These studies are encouraging but limited by utilization of the inducible *Taz*-deficient mouse. In this model, *Taz* knockdown is significant, but animals still have detectable levels of Taz protein expression in some tissue types.

Unlike *Taz* knockdown, global *Taz*-knockout (KO) in mice causes embryonic lethality and severe perinatal mortality with only about 20% of live-born *Taz*-KO pups surviving to adulthood [25]. Treatment of 1-day-old *Taz-*KO pups with an AAV9 vector encoding human *TAZ* under control of the CAG promoter, a strong, ubiquitous synthetic promoter containing a cytomegalovirus immediate early gene enhancer element (AAV9-CAG-hTAZ), significantly improved survival to the age of weaning. This treatment also prevented the development of cardiac fibrosis and abnormal MLCL/CL ratios in cardiomyocytes, indicating that *TAZ* gene replacement can prevent the development of BTHS. Treatment of 3-month-old *Taz*-KO mice that survived to the age of weaning resulted in progressive improvement in cardiac function and MLCL/CL ratios in cardiomyocytes and prevented the development of cardiac hypertrophy, extensive fibrosis and left ventricle dilation [25]. These results indicate that *TAZ* gene replacement can not only prevent development of the BTHS phenotype but can also correct an already existing phenotype.

Cardiac-specific *Taz* knockout (*Taz-*CKO) mice develop cardiomyopathy that does not impact overall survival compared to wild-type mice [25]. Treatment of 20-day-old, pre-symptomatic *Taz-*CKO mice with AAV9-CAG-hTAZ prevented the development of cardiomyopathy. Treatment of 2-month-old *Taz-*CKO mice with established cardiomyopathy resulted in normalized cardiac function and MLCL/CL ratios in cardiomyocytes, and reduced cardiac fibrosis, again showing that *TAZ* gene replacement can correct an established phenotype [25]. This study also shows that dosing is crucial to efficacy. In the *Taz*-CKO model, a higher dose of AAV9-CAG-hTAZ corresponded to a higher percentage of transduced cells and greater improvements. This will likely be even more important in BTHS patients who will have established disease when receiving therapy.

### Friedreich ataxia

Friedreich ataxia (FRDA) is caused by a GAA trinucleotide expansion in intron 1 of *FXN*, which encodes the frataxin protein (FXN) [26]. This GAA expansion causes a deficiency in *FXN* transcript levels that ultimately leads to deficiency of the FXN protein [26]. Disease severity is related to the length of the GAA repeat [26]. FXN is a small mitochondrial protein involved in assembly of iron-sulfur (FeS) clusters in the mitochondrial matrix (Fig. 2) [27]. Fe-S clusters are cofactors for several ETC complexes and other enzymes involved in the citric acid cycle [27]. FXN protein deficiency ultimately results in a secondary deficiency of FeS cluster proteins and reduced mitochondrial ATP production [27].

FRDA affects the central and peripheral nervous systems, the heart and the pancreas (Fig. 1) [26]. Symptoms usually develop between the ages of 10 and 16 years and include difficulties in walking, balance and speech [26]. Symptoms are progressive and usually necessitate wheelchair use when a patient is in his or her thirties [28]. Many patients develop swallowing difficulties (dysphagia) and eventually require tube feeding [26]. Cardiomyopathy is extremely common and the direct cause of death in about 60% of FRDA patients [29]. Most patients live about 40–50 years [29]. Standard heart failure drugs and physical and occupational therapy are common in FRDA management [26]. A variety of experimental therapies are under investigation for FRDA patients including protein replacement therapy [30], epigenetic-modifying drugs like HDAC inhibitors [31], and synthetic antisense oligonucleotides to increase FXN protein expression [32]. The variety of experimental pharmacological FRDA therapies being tested is comprehensively summarized by Clay et al [33].

Global knockout of *Fxn* is embryonic lethal in mice [34]; therefore, conditional knockout (CKO) models are commonly used to study FRDA. The *Mck* (muscle creatine kinase) conditional knockout mouse (*Mck*-CKO) has conditional knockdown of *Fxn* in skeletal and cardiac muscle [35]. These animals develop cardiac hypertrophy that eventually progresses to dilated cardiomyopathy [35]. Treatment of pre-symptomatic, 3-week-old *Mck*-CKO mice with AAVrh10-CAG-hFXN prevented development of cardiomyopathy and prevented premature death [36]. The AAVrh10 serotype transduces various organs including the peripheral and central nervous systems and the heart. The hearts of treated mice were histologically normal, and hFXN protein expression was detectable even 32 weeks after treatment [36]. AAVrh10-CAG-hFXN treatment of 7-week-old symptomatic mice improved cardiac function to normal levels and prevented the development of severe cardiac fibrosis [36]. Survival was greatly improved, but some treated animals eventually developed muscle atrophy and kyphosis, leading to death between 18 and 22 weeks of age [36]. This suggests that mitochondrial myopathy can develop if *Mck*-CKO mice survive cardiomyopathy, and that AAVrh10-CAG-hFXN does not sufficiently transduce muscles to prevent this deficit from appearing. This work provides strong evidence that *FXN* gene replacement by AAV vector-based gene therapy could provide significant therapeutic benefit to patients, even those with advanced disease. The phenotype of *Mck*-CKO mice is considered more severe and rapidly progressive than that of human FRDA, further indicating the power of a *FXN* gene replacement approach. An AAVrh10-hFXN gene therapy is currently under development for clinical testing by Voyager Therapeutics [37]. However, it is important to note that high-level overexpression of hFXN in *Mck-*CKO mice leads to cardiac toxicity [38], illustrating the importance of titrating gene therapy to the lowest dose necessary to achieve therapeutic efficacy, either through altering the dose or choosing a promoter or capsid to limit expression to affected tissue.

Another mouse model of FRDA focuses on the sensory neuropathy and ataxia associated with the disease. This mouse model has conditional knockout of *Fxn* in parvalbumin positive cells (*Pvalb*-CKO mice) [39]. *Pvalb*-CKO mice have a strong reduction in Fxn protein expression in cervical, lumbar, and thoracic dorsal root ganglia (DRG), and in the cerebellum, which leads to loss of Purkinje cells, gait abnormalities, and loss of sensorimotor reflexes [39]. *Pvalb*-CKO mice develop tremors that progress to epileptic seizures leading to death at around 21 weeks of age [39]. Treatment of 3.5-week-old pre-symptomatic *Pvalb*-CKO mice with AAV9-CAG-hFXN partially protected against coordination deficits, development of sensory neuropathy and abnormal mitochondria structure [39]. However, it did not prevent tremors from developing, and treated mice died of epileptic seizures at the same ages as those of untreated animals [39].

Seven-and-a-half-week-old symptomatic *Pvalb*-CKO mice treated with simultaneous intravenous and intracranial injections of AAVrh10-CAG-hFXN and AAV9-CAG-hFXN, respectively, showed rapid improvement in peripheral coordination and sensorimotor reflexes [39]. Treated animals had normal sciatic nerves, no loss of DRG neurons, and increased numbers of Purkinje cells [39]. Despite the improvement in symptoms and pathology, treated animals still developed significant tremors that prompted euthanasia before death due to epilepsy [39]. These results suggest that gene replacement therapy can correct the neuropathy associated with FRDA. However, it is important to note that the *Pvalb-*CKO mouse model has limitations as a model for FRDA. Epilepsy and seizures are very rare in human FRDA patients. The primary cause of death in these patients is cardiomyopathy [40]. Though this study did not show an increase in survival, treated animals showed improvement in ataxia and motor reflexes, the symptoms that most significantly impact a patient’s quality of life. Even if lifespan is not impacted, a treatment that improves these symptoms could greatly benefit FRDA patients.

Another FRDA model has conditional knockdown of *Fxn* in neuron-specific enolase (NSE) positive cells (*Nse*-CKO), leading to lack of Fxn protein in neurons, heart, muscle, kidney and liver [35]. *Nse*-CKO mice develop cardiac hypertrophy and neurological symptoms that lead to a decreased lifespan [35]. Pre-symptomatic *Nse*-CKO mice treated at the age of 5–9 days with AAV9-CB-hFXN (human FXN under control of the CMV enhancer/β-actin (CB) promoter) had a longer average lifespan than untreated mice (135 days compared to 35 days) [41]. Treated animals had reduced cardiac hypertrophy, modest improvement in cardiac function and improved activity levels [41]. AAV9-CB-hFXN treatment also increased the survival of *Mck*-CKO mice from a mean of 71 days to a mean of 194 days, prevented the development of cardiac hypertrophy and improved cardiac function [41].

An inducible mouse model of *Fxn* knockdown (FRDAkd) allows researchers to temporally control *Fxn* expression and allows for re-expression of *Fxn* after knockdown [42]. FRDAkd mice with *Fxn* knocked down have decreased survival and muscle strength and develop cardiomyopathy [42]. Re-expression of *Fxn* in FRDAkd animals already showing symptoms results in significantly improved survival, cardiac function and motor performance [42]. Though not directly testing gene replacement therapy, this data supports the hypothesis that increasing expression of the *Fxn* gene, as gene therapy aims to do, can lead to meaningful recovery even in established pathology.

### NDUFS4, NDUFS3, and SURF1 deficiency-related Leigh syndrome

Leigh syndrome (LS) is the most common presentation of mitochondrial disease in children [43]. LS-causing mutations have been identified in over 75 different genes in the nuclear and mitochondrial genomes [5]. About 10–20% of LS patients have a mutation in their mitochondrial DNA [5]. LS is a serious and progressive neurodegenerative disease characterized by the development of areas of spongiform degeneration throughout the brain, particularly in the brainstem and basal ganglia [43]. This degeneration leads to ataxia, ophthalmologic abnormalities, hearing loss, cardiomyopathy, hypotonia, dystonia, muscle weakness and gastrointestinal problems (Fig. 1) [43, 44]. In about 80% of cases, symptoms begin in infancy or early childhood and lead to death by 3 years of age [44]. Respiratory insufficiency due to brain stem degeneration and muscle weakness is the leading cause of death [5]. LS genes code for ETC components, ETC assembly components, pyruvate dehydrogenase complex components, mitochondrial transcription/translation proteins, or proteins involved in thiamine metabolism (Fig. 2) [44]. Currently, only supportive therapies, such as drugs to manage cardiomyopathy and lactic acidosis, are available [45]. Some patients with LS caused by pyruvate dehydrogenase complex (PDHC) deficiency showed symptom improvement on a ketogenic diet [46]. Pharmacological inhibition of the mTOR pathway has shown positive results in a mouse model of NDUFS4 deficiency-related LS (the *Ndufs4* knockout mouse) [47] and a single patient [48].

*Ndufs4* knockout (*Ndufs4*-KO) mice develop a severe and progressive encephalomyopathy that becomes apparent at about 40 days of age [49]. The average survival of the mutant animals is around 55 days [49]. Newborn *Ndufs4-*KO pups receiving either intravenous (IV) or intracerebral ventricular (ICV) injection of AAV9-CMV-hNDUFS4 (human *NDUFS4* under control of the strong, constitutive cytomegalovirus (CMV) promoter) alone did not show increased longevity compared to untreated animals [49]. However, newborn *Nudufs4-*KO pups treated with both IV and ICV injections showed significant improvement in ataxia and overall survival, with increased mitochondrial complex I activity in brain, muscle and heart tissues [49]. These results suggest that treating both brain and peripheral tissues is critical for prevention of NDUFS4 deficiency-related LS [49].

Reynaud-Dulaurier et al*.* and Silva-Pinheiro et al. found that a single dose of AAV-PHP.B-hNDUFS4 therapy administered at 1 month old was effective at delaying disease progression in adult *Ndufs4-*KO mice [50, 51]. AAV-PHP.B is a laboratory developed mutant of AAV9 with enhanced ability to cross the blood–brain barrier and transduce neuronal and glial cells following systemic vector delivery [20]. In both studies, *Ndufs4-*KO animals treated with gene therapy (AAV-PHP.B-CAG-hNDUFS4 in Reynaud-Dulaurier et al. and AAV-PHP.B-CMV-hNDUFS4 in Silva-Pinheiro et al.) showed increased NDUFS4 protein expression in the brain, improved motor function, increased weight, increased complex I activity in brain mitochondria, and decreased gliosis [50, 51]. Fifty percent of *Ndufs4-*KO mice treated with AAV-PHP.B-CAG-hNDUFS4 survived to 250 days, while untreated *Ndufs4-KO* animals died at a median of 54 days [50]. AAV-PHP.B-CMV-hNDUFS4 treatment increased survival of *Ndufs4-*KO animals from 55 to 100 days, and 30% of treated animals survived to over 1 year [51]. These studies indicate that there may be a window where successful gene replacement treatment is possible, as treatment of *Ndufs4*-KO mice as adults results in prolonged survival.

Mutations in *NDUFS3* have also been associated with LS [52]. Like NDUFS4, NDUFS3 is a component of electron transport chain complex I (Fig. 2) [53]. Mice with *Ndufs3* knocked out in skeletal muscle (*Ndufs3*-smKO) experience progressive myopathy characterized by a decline in motor coordination and overall activity [54]. Complex I activity is significantly decreased in the skeletal muscles of mutant animals [54]. Mutant mice also have significant serum lactic acidosis [54]. Systemic treatment with AAV9-CMV-Ndufs3 at 15–18 days of age prevented the development of myopathy and decline of complex I activity, normalized serum lactate levels and significantly extended survival [54].

Remarkably, AAV9-CMV-Ndufs3 treatment resulted in reversal of the *Ndufs3*-smKO phenotype when administered to 2-month-old symptomatic animals [54]. At the time of injection, mutant mice showed significantly worse performance in strength and motor coordination than wild-type littermates [54]. Three months after injection, there was no difference in strength/coordination test performance between treated *Ndufs3*-smKO mice and wild-type mice [54]. Complex I activity in skeletal muscles returned to near wild-type levels and lactic acidosis was reversed [54]. These results suggest that recovery from established myopathy may be possible. However, no survival data was presented, so it is not clear if these improvements result in a meaningful survival increase.

*SURF1* encodes the surfeit locus protein 1 (SURF1) protein. SURF1 is localized in the inner mitochondrial membrane and is a component of the mitochondrial translation regulation assembly intermediate of cytochrome c oxidase (MITRAC) complex (Fig. 2) [55]. The MITRAC complex regulates cytochrome c oxidase (COX or complex IV) assembly [55]. Mutations in *SURF1* can result in defects in complex IV (CIV) assembly which ultimately leads to CIV dysfunction [56]. Around 200 cases of *SURF1-*associated LS have been reported in the literature [5]. Curiously, unlike LS patients and *Ndufs4*-KO and *Ndufs3-*smKO mice, *Surf1* knockout (KO) mice do not develop neurodegeneration or myopathy [57]. *Surf1-*KO mice have a prolonged lifespan compared to WT and heterozygous individuals [57]. The only observable pathology in *Surf1*-KO mice is modestly decreased CIV activity and slightly elevated blood lactate [57].

Ling et al. [58] treated 1 month old *Surf1-*KO mice with self-complimentary AAV9 carrying human *SURF1* under control of the CB promoter. Unlike the native AAV genome, self-complementary vectors are double stranded, making synthesis of the second strand unnecessary [59]. A self-complementary vector, however, can only carry half the genetic material of a single-stranded vector (about 2.4 kb vs 4.7 kb). Ling et al. [58] chose to use self-complimentary AAV due to the relatively small size of the *SURF1* cDNA and CB promoter and some evidence that self-complimentary AAV9 vectors result in a higher number of transduced cells [60]. scAVV9-CB-SURF1 was administered by intrathecal (IT) injection or both IT and IV injection. *Surf1-*KO mice treated with the highest intrathecal dose of scAAV9-CB-hSURF1 had a statistically significant, though very modest, increase in CIV activity in the cerebrum, cerebellum, liver and muscle compared to wild-type animals [58]. A combination of IV and IT delivery of the therapeutic vector did not result in increased CIV activity compared to IT treatment alone [58]. This study did not find a difference in resting blood lactate between wild-type and mutant mice, but at 10 months of age, *Surf1-*KO mice did experience a significantly greater increase in blood lactate levels after running on a treadmill until exhaustion than wild-type mice [58]. This increase in blood lactate following exhaustive exercise was reduced in *Surf1-*KO mice treated with scAAV9-CB-hSURF1 [58].

These data provide modest evidence that delivery of *SURF1* by AAV can result in functional changes. However, enthusiasm is significantly limited by the Surf1-KO mouse failing to recapitulate the human disease in a meaningful way. CIV activity was increased in AAV treated *Surf1-*KO animals, but this increase was very modest, and it is unknown if it is sufficient to provide clinical benefit. The majority of LS patients experience increased blood lactate [5], so it is encouraging that blood lactate can be affected by scAAV9-CBh-SURF1 therapy. Results from multiple LS studies discussed above suggest that serum or blood lactate level could be a potential biomarker for monitoring efficacy of a treatment.

It must be reiterated that as LS can be caused by mutations in many other genes beyond *NDUFS4*, *NDUFS3*, and *SURF1*. Therefore, gene replacement therapy for a LS patient would need to deliver the wild-type version of the gene that causes the disease in that particular patient. Given the extensive genetic diversity of LS, this would require a personalized therapy for each individual patient.

### Ethylmalonic encephalomyopathy

Ethylmalonic encephalopathy (EE) is an autosomal recessive mitochondrial disease caused by a mutation in the ethylmalonic encephalopathy protein 1 (*ETHE1)* gene [61]. EE symptoms include severe and progressive hypotonia, developmental delay, intellectual disability, seizures, spasticity, diarrhea and microvascular damage (Fig. 1) [62]. Biochemically, the disease is characterized by persistent lactic acidemia, elevated plasma levels of acylcarnitine and acylglycines, and elevated urinary excretion of ethylmalonic acid (EMA) [61]. Symptoms develop in infancy, and patients typically do not live past 10 years of age. *ETHE1* encodes a mitochondrial sulfur dioxygenase (SDO) involved in the detoxification of H_2_S (Fig. 2) [62]. H_2_S is produced by the metabolism of amino acids containing sulfur and by anaerobic bacteria in the large intestine [61]. At elevated levels, H_2_S damages the vascular endothelium [63] and inhibits enzymes such as cytochrome c oxidase (COX) and short chain acyl-CoA dehydrogenase (SCAD), leading to impaired mitochondrial function in the brain and muscles of EE patients [64–66].

It is hypothesized that reducing H_2_S levels will prevent further damage to the brain, intestinal mucosa and vasculature. Treatment with the antibiotic metronidazole and N-acetylcysteine, a precursor to H_2_S-buffering glutathione, lowers serum thiosulfate levels (an indicator of H_2_S levels) in *Ethe1* knockout (*Ethe1*-KO) mice and in human patients, with patients showing some clinical improvement in gastrointestinal symptoms [67–69]. Liver transplant has been attempted in three patients, with the hypothesis that a non-diseased liver could provide sufficient blood clearance of H_2_S which would lead to improved symptoms [70]. Acetylcysteine and metronidazole have few side effects, but liver transplant is a serious intervention requiring life-long immunosuppression. Clearly, other treatment options are needed for these patients.

*Ethe1*-KO mice have a biochemical phenotype similar to EE patients, with elevated levels of urine EMA and thiosulfate, and elevated serum acylcarnitines compared to those of wild-type littermates [62]. H_2_S levels in brain, liver and muscle are elevated, leading to decreased COX activity in these tissues [62]. *Ethe1-*KO mice have arrested growth, decreased motor activity and a significantly shortened lifespan compared to wild-type mice [62]. Treatment of 21-day-old *Ethe1*-KO mice with AAV8-TBG-hETHE1 (AAV8 is a hepatotropic AAV serotype and the thyroxine-binding globulin gene enhancer (TBG)-promoter drives liver-specific expression of the transgene) improved survival, increased motor activity and increased COX activity in the brain and skeletal muscle [71]. The length of survival in treated animals correlated with plasma thiosulfate levels, with individuals with lower thiosulfate levels surviving the longest [71]. Thiosulfate levels also correlated with SDO activity and *ETHE1* gene copy number in hepatocytes [71]. This indicates that efficacy of hepatocyte transduction directly correlates with the effectiveness of the treatment. Thiosulfate levels in skeletal muscle were significantly lowered in mice treated with AAV8-TBG-hETHE1, demonstrating that clearance of systemic H_2_S will result in clearance of H_2_S from tissues [71]. This suggests that a liver-targeting gene therapy strategy could be effective for EE patients and that plasma thiosulfate levels may be a useful biomarker.

### Three mitochondrial DNA depletion syndromes

Mitochondrial DNA depletion syndromes (MDS) are autosomal recessive diseases characterized by a severe reduction in mitochondrial DNA content [72]. This leads to inadequate synthesis of crucial ETC components and ultimately inadequate energy production [72]. Some MDS-causing genes are associated with mtDNA replication, dNTP synthesis, or mitochondrial fission/fusion [72]. Clinically, MDS have a variable presentation that is broken down into four subcategories based on the affected organ systems: myopathic, hepatocerebral, encephalomyopathic and neurogastrointestinal [72].

#### MNGIE

Mitochondrial neurogastrointestinal encephalomyopathy (MNGIE) is the most common neurogastrointestinal MDS [73]. It is caused by mutations in the thymidine phosphorylase (*TYMP*) gene [73]. *TYMP* encodes thymidine phosphorylase (TP), an enzyme that plays a key role in pyrimidine salvage pathways [73]. TP catalyzes the reversible conversion of thymidine (dThd) and deoxyuridine (dUrd) to their bases thymine and uracil [73]. Mutations in *TYMP* cause deficiency of TP protein. TP protein deficiency results in an accumulation of dThd and dUrd that leads to mtDNA depletion, which ultimately results in mitochondrial dysfunction (Fig. 2) [74]. Patients typically develop symptoms of gastrointestinal dysmotility, such as vomiting, diarrhea, and difficulty swallowing, in their late teens to early twenties [73] (Fig. 1). Neurological symptoms include ptosis, external ophthalmoplegia, hearing loss and peripheral neuropathy [73] (Fig. 1). The average lifespan of MNGIE patients is 37 years, with death usually resulting from gastrointestinal complications [73]. A number of different experimental therapeutic options are being explored, including dialysis [75], liver transplantation [76, 77], hematopoietic stem cell (HSC) transplantation [78], and enzyme replacement therapy [79]. Patients who receive dialysis or those who have undergone successful liver or HSC transplant showed decreased plasma thymidine levels and modest improvement or stabilization of gastrointestinal symptoms [75, 76, 78, 80]. However, HSC transplantation is associated with high mortality in this population [78]. There is a clear need for better options for patients suffering from this disabling condition.

Scientists have approached gene therapy for MNGIE from two angles: lentiviral vector-mediated HSC therapy and AAV vector-based gene replacement therapy. Preclinical studies to investigate these two approaches used the *Tymp/Upp1* double knockout (*Tymp/Upp1*-DKO) mouse [81]. *Upp1* encodes uridine phosphorylase (UP), which catalyzes the conversion of deoxyuridine to uracil [81]. Unlike human UP, murine UP can also convert thymidine to thymine, requiring both *Tymp* and *Upp1* knockdown to create a mouse model truly lacking thymidine phosphorylase activity [81]. In this model, serum levels of dThd and dUrd are significantly elevated and brain mitochondria show defective electron transport chain function [81]. *Tymp/Upp1-*DKO mice also have evidence of white matter changes detectable by MRI [81]. Several groups have preclinical data showing the potential efficacy of transplanting *Tymp/Upp1*-DKO mice with lentiviral vector-transduced HSCs [82–84]. Although treated animals in these studies showed decreased serum thymidine levels, the transplant procedure itself shortened lifespan. An alternative option is AAV vector-based gene replacement therapy.

Adult *Tymp/Upp1*-DKO mice treated with a hepatotropic AAV vector encoding human *TYMP* under control of a liver-specific promoter (AAV8-TBG-hTYMP) had sustained lowering of dThd and dUrd plasma levels for 15 weeks following treatment before rising back to pre-treatment levels in the lowest tested dose [85]. In animals treated with the highest dose of AAV8-TBG-hTYMP, dThd and dUrd plasma levels remained below the levels of the wild-type control even 22 months post-treatment [85]. Another study exploring AAV8 vector-mediated delivery of *TYMP* found that *hTYMP* expression driven by a liver-specific promoter (α-1-antitrypsin [AAT], human thyroxine binding globulin [TBG], or hybrid liver-specific promoter [HLP]) was more effective than a ubiquitous promoter (phosphoglycerate kinase, PGK) at achieving a sustained decline in plasma dThd levels [86]. Even at the lowest tested dose, all mice treated with an AAV vector had dThd levels at or close to the levels in the wild-type controls 4 weeks after treatment [86]. This effect was sustained for 34 weeks in mice treated with AAV8 vector encoding *hTYMP* driven by a liver-specific promoter [86]. At 34 weeks post-treatment, robust TP enzyme activity was detectable in the liver [86]. In animals receiving the highest dose of AAV8-AAT-hTYMP, TP enzyme activity was detected in the brain and gastrocnemius muscle. Both tissues also showed dThd levels lower than the levels seen in tissue from wild type animals [86].

Exposing *Tymp/Upp1*-DKO mice to thymidine and deoxyuridine in their drinking water caused elevated dThd and dUrd levels and a more severe phenotype, with older animals showing signs of decreased muscle strength and motor coordination [87]. Though dThd and dUrd levels were increased in the tissues of *Tymp/Upp1*-DKO mice exposed to exogenous nucleosides, no gastrointestinal pathology developed [87]. AAV vector-mediated delivery of *hTYMP* under a liver-specific promoter (AAT, TBG, HLP) to dThd and dUrd exposed mice 8–11 weeks of age resulted in decreased nucleoside levels in tissue and serum, and modest improvement in motor function as measured by rotarod performance at 25 weeks of age [88]. AAV gene therapy also resulted in a modest reduction of ventricular volume in treated animals compared to untreated animals at 84 weeks old [88]. This data suggests that reduction of nucleoside levels can lead to symptom improvement, but the observed therapeutic effects were modest. Nucleoside levels will make a useful biomarker for monitoring progression of MNGIE and response to therapy.

The studies discussed above provide strong preclinical rationale for testing liver targeted AAV gene therapy in MNGIE patients. The major limitation is the mouse model. *Tymp/Upp1-DKO* mice, even those experiencing chronic dThd and dUrd exposure, do not display the characteristic gastrointestinal phenotype seen in human patients. The lack of symptoms in mice is perhaps due to the short lifespan of the animals, or the fact that dThd levels are only moderately elevated. Human MNGIE patients have dThd levels up to 350-fold higher than healthy subjects [89], but the *Tymp-Upp1-*DKO mouse model shows only about a fourfold increase compared to wild-type animals [85, 86]. Chronic nucleoside exposure increases the plasma levels of *Tymp-Upp1-*DKO mice 30-fold over wild-type mice [87], but that is still not sufficient to induce gastrointestinal pathology, making it difficult to ascertain if the maintenance of lowered dThd levels by gene therapy will lead to meaningful improvement of gastrointestinal symptoms.

#### MPV17

About half the cases of hepatocerebral MDS are caused by mutations in *MPV17* [90]. MPV17 protein is localized to the inner mitochondrial membrane, but its function is not clearly understood [91]. Research indicates that *MPV17* mutations lead to mitochondrial deoxynucleotide insufficiency (Fig. 2) [92]. MPV17 hepatocerebral MDS symptoms present as hypoglycemic crises and rapidly progressive liver dysfunction leading to cirrhosis and liver failure, usually within the first year of life [72] (Fig. 1). Neurological symptoms are mild at first and progress in those patients that survive early hepatic failure (Fig. 1). Currently, the only therapeutic interventions are supplementing the diet with uncooked cornstarch to reduce hypoglycemic episodes [93], and liver transplant [72]. Liver transplant is an incredibly risky procedure in this patient population; of the 17 patients documented to have received transplants, 60% died in the post-transplant period [94].

The *Mvp17*-knockout (*Mpv17*-KO*)* mouse model shows almost complete depletion of mtDNA in the liver and some depletion of mtDNA in the skeletal muscle [95]. However, despite elevated serum markers of liver dysfunction are some changes in liver histology, these mice do not develop overt liver dysfunction [95]. Mitochondrial DNA transcript levels are elevated in *Mvp17* knockout hepatocytes, possibly through a mechanism to compensate for the depleted levels of mtDNA. The primary phenotype in this model is development of kidney dysfunction in the form of focal segmental glomerulosclerosis by 18 months of age, a phenotype not seen in humans [95].

*Mpv17*-KO mice fed a ketogenic diet for 2 months develop liver cirrhosis and liver failure and have extremely elevated plasma alanine aminotransferase (ALT) and aspartate transaminase (AST) levels, indicating liver dysfunction [90]. Treatment of 2-month-old *Mpv17*-KO mice with an intravenously injected AAV8 vector encoding human *MPV17* under control of a liver-specific promoter (AAV8-TBG-hMPV17) lead to decreased serum AST and ALT levels, suggesting correction of liver damage [90]. In treated mice, hepatocellular mtDNA copy number was increased to levels close to those of untreated control animals [90]. The activity of ETC complexes also increased in the hepatocytes of treated mice, indicating that restoration of mtDNA levels leads to functional improvement [90]. Treatment with AAV8-TBG-hMPV17 3 weeks prior to ketogenic diet protected mutant animals from developing cirrhosis and fibrosis [90]. Hepatocellular mtDNA copy number was significantly elevated in mice treated before beginning ketogenic diet, though it did not reach the levels seen in heterozygous mice [90]. This increase in mtDNA copy number was sufficient to significantly increase the expression of ETC complex transcripts [90]. AAV8-TBG-hMPV17 treatment protected against additional liver damage when administered to *Mpv17*-KO mice with liver damage from ketogenic diet [90]. Knockout mice were fed a ketogenic diet for 1 month before the AAV vector treatment and continued the diet for 1 month following treatment [90]. Liver damage was less extensive in treated animals and hepatocellular mtDNA copy number was increased leading to an increase in ETC transcripts compared to untreated mice [90]. Post-ketogenic diet treated *Mpv17-*KO mice had less extensive liver damage than untreated mice, but treated mice continued to lose weight at the same rate as untreated mice, indicating that AAV vector-based therapy was not able to slow clinical decline [90]. Likely, post-ketogenic diet AAV vector-based therapy prevents development of further damage but does not correct existing damage [90]. This underscores the importance of careful consideration of the timing of gene therapy during the course of disease progression.

#### TK2-deficiency

Thymidine kinase 2 (TK2) deficiency is caused by homozygous recessive mutations in the *thymidine kinase 2 (TK2*) gene [96]. Like *TYMP*, *TK2* is part of the mitochondrial pyrimidine salvage pathway, which is critical for maintaining mtDNA levels [97]. TK2 is a mitochondrially localized protein that phosphorylates deoxycytidine (dC) and deoxythymidine (dThd) to generate deoxycytidine monophosphate (dCMP) and deoxythymidine monophosphate (dTMP). Defects in TK2 function lead to mtDNA depletion (Fig. 2) [97]. TK2 deficiency has a heterogenous clinical presentation and spectrum of symptoms. Patients with the severe form of TK2 deficiency show early-onset and rapidly progressive myopathy, usually leading to respiratory failure within 2 years [96]. CNS involvement is not uncommon [96]. Adult-onset TK2 deficiency is generally less severe with a slower disease progression [96]. The most common cause of death is respiratory insufficiency [96]. Other common symptoms are hearing loss, dysphagia, sensory neuropathy and chronic progressive external ophthalmoplegia (CPEO) [96]. (Fig. 1) TK2 deficiency often eventually necessitates the need for respiratory support, feeding tubes, and mobility devices such as wheelchairs. Currently, supplementation of dC and dThd is being explored as a potential therapeutic option [98].

Mice homozygous for a disease causing mutation (*Tk2*-/- mice) develop a mitochondrial myopathy similar to that observed in early onset human TK2 deficiency [99]. *Tk2-/-* mice develop normally until 10 days old, after which the mutant animals show decreased growth and locomotor activity, tremor and abnormal gait [99]. This rapidly developing phenotype results in death at around P14 [99]. Tk2 activity is significantly reduced in multiple tissue types and corresponds with decreased levels of mtDNA [99]. Brain mitochondria have decreased complex I and IV activity and protein levels [99]. Deoxycytidine and dThd supplementation increased the survival of *Tk2-/-* mice up to threefold [100].

Lopez-Gomez et al. treated *Tk2-/-* mice with AAV9 carrying human *TK2* under control of the CB promoter [101]. *Tk2-/-* mice received one of two doses of AAV9-CB-hTK2 at P1 or a low dose of AAV9-CB-TK2 at P1 and a low dose of AA2-CB-TK2 at P29 [101]. All treatments extended survival, with the combination AAV9/AAV2 treatment resulting in the greatest increase [101]. It should be noted that the total dose of AAV9/AAV2 treated mice was less than the AAV9-alone treated animals. Adding dC/dThd therapy beginning at P21 further increased the survival of AAV9/AAV2-hTK2 treated mice [101]. Mice treated with this AAV9/AAV2/deoxynucloside combination had the most improved growth of all tested treatments, though no treatment lead to *Tk2-/-* animals achieving the same adult weight as wild-type animals [101]. High dose AAV9, AAV9/AAV2, and AAV9/AAV2/deoxynucleoside treated mice showed the same performance in motor function tests as wild-type mice at 60 and 90 days of age [101]. Levels of mtDNA were increased to close to wild-type levels in the brain, liver, heart and intestines in treated mice [101]. mtDNA levels were also increased in the skeletal muscle of treated animals, but not to wild-type levels [101]. Interestingly, brain TK2 activity was only increased to about 50% of wild-type but still resulted in normal levels of mtDNA in this tissue [101].

This study demonstrates that gene therapy can greatly improve not only the survival, but also the motor function of *Tk2-/-* mice. It remains unknown if gene therapy rescues the gliosis observed in the brains and spinal cords of *Tk2-/-* mice in other studies. This study shows that the efficacy of gene therapy can be enhanced when combined with other therapeutic options, in this case nucleoside supplementation. It is likely that the best clinical management of mitochondrial diseases will ultimately be achieved with combination therapy, rather than any individual therapy.

### SLC25A46-related disease

The SLC25A46 protein is an outer mitochondrial membrane lipid transporter that transports lipids from the endoplasmic reticulum to the mitochondria (Fig. 2) [102]. It promotes mitochondrial fission through its interactions with mitofusin 2 (MFN2), OPA1 mitochondrial dynamin like GTPase (OPA1), and the mitochondrial contact site and cristae organizing system (MICOS) (Fig. 2) [102]. Mutations in *SLC25A46* lead to mitochondrial hyperfusion, abnormal cristae structure, and abnormal mitochondrial architecture [102]. *SLC25A46* gene mutations have been identified in patients with Leigh syndrome [102], autosomal recessive cerebellar ataxia [103], progressive myoclonic ataxia with optic atrophy [104], Charcot-Marie Tooth syndrome 2 [105], and lethal pontocerebellar hypoplasia [106] (Fig. 1). Mice homozygous for a loss-of-function mutation in *Slc25a46* display a similar phenotype to that of human patients: ataxia, muscle weakness, optic atrophy and cerebellar hypoplasia [107]. These symptoms appear about 2 weeks after birth and the majority of mutant animals die by 5 weeks of age [107].

Treatment of 2-day-old *Slc25a46* mutant mice with a highly brain penetrant AAV capsid (AAV-PHP.eB) encoding mouse *Slc25a46* under control of a ubiquitous promoter (CMV) lead to significantly prolonged lifespan and improved coordination and strength [108]. The cerebella of treated animals had reduced numbers of inflammatory cells (astrocytes and microglia) and increased numbers of Purkinje neurons [108]. Treated mice had more retinal ganglion cells and thicker axons in the optic nerves compared to untreated animals [108]. Since no functional vision experiments were conducted, it is unclear if these changes would lead to meaningful vision improvement. Unlike untreated animals, the sciatic nerves of treated animals had normal-appearing mitochondria, little evidence of demyelination and improved conduction [108]. Overall, these results indicate that gene replacement via AAV vector can partially prevent the development of the phenotype associated with *SLC25A46* mutations. It is unclear, though, if gene replacement therapy administered to symptomatic individuals would provide any therapeutic benefit.

### Leber hereditary optic neuropathy

Ophthalmological diseases are uniquely suited to treatment with gene therapy as the eye is easily accessible, compartmentalized, and immune privileged. Therapy can be directly administered, reducing dosage and potential systemic side effects including the development of anti-therapeutic antibodies [109]. The first gene therapy product approved by the FDA was voretigene naparvovec (Luxturna) for the treatment of Leber congenital amaurosis (LCA) type 2*,* an inherited retinal disease that causes progressive vision loss [110].

Gene therapy options are currently being investigated for Leber hereditary optic neuropathy (LHON), a mitochondrial retinopathy (Fig. 1). In LHON patients, vision loss usually begins in the teenage years or young adulthood [111]. Disease progression is very rapid and visual acuity diminishes to 20/200 (legally blind) within 5–6 weeks of symptom onset [111]. LHON is caused by a point mutation in one of the genes that code for the components of mitochondrial NADH dehydrogenase. Mutations in the NADH dehydrogenase subunit 4 (*MT-ND4)* gene account for 70% of cases [112]. These mutations lead to dysfunction of complex I of the electron transport chain (Fig. 2) [112]. How complex I dysfunction leads to loss of retinal ganglion cells (RGCs) has yet to be determined [112]. Currently, there is only one approved treatment for LHON–idebenone, a short-chain benzoquinone with anti-oxidant properties and the ability to act as an electron carrier in the ETC [113]. Idebenone treatment protects against color vision loss and deterioration of vision in LHON patients [113].

Unlike the other diseases discussed in this review, LHON is caused by a mutation in mtDNA. There are several challenges in applying gene therapy to a disease caused by a mtDNA mutation. Each cell has many mitochondria (a range of 80–2000, depending on cell type) and each mitochondrion has 2–10 copies of the mitochondrial genome [114]. This likely means that an effective gene therapy would require multiple copies of the therapeutic gene. The genetic code of the mitochondrial genome is different from the nuclear genetic code, and mitochondrial genes are transcribed and translated into their protein products within the mitochondria [115]. Thus, a gene therapy product must be capable of crossing the cell membrane and then entering the mitochondria or the therapeutic gene would need to be recoded for nuclear expression followed by eventual mitochondrial import.

A gene therapy product for LHON, GS010 (Lumevoq) by GenSight Biologics is being evaluated in multiple Phase III studies in the United States and was submitted for European approval in the fall of 2020. GS010 is an AAV2 vector-based therapy product encoding wild-type *MT-ND4* with a mitochondrial targeting sequence (MTS) that allows ND4 protein to be transported into mitochondria [116]. Patients in the REVERSE (NCT02652780) and RESCUE (NCT02652767) trials received an intravitreal injection of Lumevoq in one eye [116, 117]. Patients in both trials showed bilateral improvement in visual acuity [116, 117].

Another approach to targeting therapies to the mitochondria is to modify the AAV capsid. The Guy laboratory at the University of Miami added an MTS to the AAV2 capsid, facilitating import of AAV2 capsids to the mitochondria [118, 119]. Specifically, the cytochrome c oxidase subunit 8 (COX8) MTS was added to the N-terminus of the AAV capsid VP2 protein [118, 119]. MTS-AAV encodes human *MT*-*ND4* under control of the mitochondrial heavy strand promoter (HSP). In cytoplasmic hybrid cell lines 100% homoplasmic for an *MT*-*ND4* mutation, MTS-AAV2-HSP-hMT-ND4-FLAG vector localized to the mitochondria [119]. FLAG tag-fused protein was detected in the mitochondria, indicating the exogenous DNA was being transcribed and translated in the mitochondria [119]. Cells with mutant *ND4* have impaired ATP synthesis [119]. Treatment with MTS-AAV2-HSP-hMT-ND4-FLAG vector increased ATP production compared to cells treated with AAV vector without an MTS [119].

Eyes of wild-type mice injected with MTS-AAV2-HSP-hMT-ND4-FLAG vector had detectable FLAG tag-fused protein expression in the mitochondria of retinal ganglion cells, and expression was retained 6 months after injection [119]. Intravitreal injection with AAV2-hMT-ND4-R340H (a disease-causing mutation) causes visual loss in mice [120]. Mice were intravitreally injected with MTS-AAV2-HSP-hMT-ND4, AAV2-HSP-hMT-ND4 or AAV2-GFP and 2 days later with AAV2-R340H-hMT-ND4 [119]. Pre-treatment with MTS-AAV2-HSP-hMT-ND4 protected mice from development of visual loss and optic nerve atrophy [119]. Mice pretreated with MTS-AAV2-HSP-hMT-ND4 had significantly higher PERG amplitudes (a measure of retinal function) and thicker optic nerves compared to animals pre-treated with AAV-GFP or AAV-HSP-hMT-ND4 [119]. This data suggests that mitochondria targeted AAV-hMT-ND4 treatment protects against vision loss caused by mutant R340H-hMT-ND4 DNA.

MTS-AAV2 has also been used to create mouse models of LHON [121]. MTS-AAV-R340W-hMT-ND4-mCherry was injected into fertilized oocytes [121]. Eyes of the resulting adult mice were examined with confocal laser-scanning ophthalmoscopy for mCherry expression in the optic nerve head [121]. Female mice with high optic nerve head expression of mCherry were backcrossed to wild-type males for 8 generations [121]. The resulting mutant “mitomice” developed progressive vision loss beginning at 3 months of age that progressed to blindness by 8 months of age [121]. Vision loss was accompanied by retinal degeneration and loss of axons and retinal ganglion cells, changes that are consistent with what happens in human LHON patients [121]. Three-month-old mitomice treated with MTS-AAV-HSP-hMT-ND4 had significantly increased PERG amplitudes 3 months post-injection [122]. This improvement was stable at 13 months post-injection, and wild-type *hMT-ND4* DNA and *hMT-ND4* transcripts were detectable at 15 months post-injection [122]. Treated mice were protected from loss of retinal ganglion cells and optic nerve atrophy [122]. These data indicate that a mitochondrially targeted AAV vector-mediated gene therapy could be a valuable therapeutic tool for LHON.

Although the MTS-AAV2 approach has been shown to be effective in rodent models as described above, important questions remain unanswered for clinical translation. The mechanism of MTS-AAV2 entry into the mitochondria is not clear. It is also unclear whether a mitochondrially targeted AAV would be more or less effective than a non-targeted AAV in a systemic mitochondrial disease like Leigh syndrome or MNGIE. Another interesting avenue would be engineering other AAV capsid serotypes to target the mitochondria. Overall, mitochondrial disease research is hampered by the lack of good models, particularly for diseases caused by mutations in the mitochondrial genome. Using MTS-AAV to introduce mitochondrial mutations into mouse zygotes could be an incredibly useful tool for creating new models [121].

## Discussion

### Challenges

AAV vector-mediated gene therapy could be a promising treatment strategy for a variety of mitochondrial diseases, including those not discussed here. Mitochondrial disease patients have few therapeutic options, and new treatment modalities, such as gene therapy, are urgently needed. Ninety percent of mitochondrial diseases are caused by mutations in a single nuclear gene, making them good targets for gene replacement therapy. As of 2021, two AAV vector-based gene therapy products (Zolgensma and Luxturna) have been approved by the FDA and regulatory agencies in other countries, and it is likely that additional AAV vector-based therapies will be approved in the coming years.

The preclinical studies presented here make use of mouse models of mitochondrial disease. A limitation of mouse models is that a mutation in the mouse homolog of a human gene does not always recapitulate the phenotype observed in humans, as seen in the *Tymp/Upp1-DKO* mouse model of BTHS and the *Surf1-KO* model of LS. Tissue specific knockdowns can be useful tools, but often do not recapitulate the full phenotype of a disease. The progression of human mitochondrial disease is rarely linear and can be difficult to predict, making it challenging to evaluate the efficacy of tested therapies. One advantage of mouse models is that the disease course is predictable and very similar between individuals, simplifying the evaluation of a tested agent’s efficacy. Another limitation is that mouse models are mostly limited to those disease caused by mutations in the nuclear genome due to the technical challenges in manipulating the mitochondrial genome. New strategies, like the “mitomouse” model of LHON, will hopefully enable the creation of mouse models carrying mutations in the mitochondrial genome and allow for preclinical evaluation of new treatment options.

The preclinical data presented here highlight the challenges associated with studying mitochondrial diseases. In addition to the rarity, the phenotypic and genetic heterogeneity of mitochondrial diseases means diagnosing these conditions is challenging and can take years—patients see an average of 8 physicians before diagnosis [123]. Though early intervention would likely provide the most clinical benefit, early diagnosis of mitochondrial disease outside the context of family history, which would allow for prenatal testing or newborn testing, is incredibly challenging. As pathogenic mutations resulting in mitochondrial disease have been identified in over 300 genes, genetic screening is impractical at this time. In patients with suspected mitochondrial disease, whole genome sequencing is useful for diagnosis [124]. The decreasing cost and increasing use of whole genome sequencing for diagnostics will hopefully reduce the time to diagnosis and enable earlier treatment for mitochondrial disease patients in the future.

A major challenge in gene therapy is timing of treatment. The majority of mitochondrial disease patients will have well established disease by the time of diagnosis, so it is critical to find therapies that can stop or slow disease progression or improve established disease. Most of the studies discussed here have shown that prevention of disease development using AAV-vector based gene replacement therapy may be possible. While this is encouraging, it is unlikely to benefit patients because most patients will already be symptomatic when they receive a genetic diagnosis. More exciting is that several of the studies discussed here suggest that gene replacement therapy using an AAV vector can result in amelioration or slowing of an established phenotype in BTHS [25], FRDA [42], NDUFS3 [54]- and NDUFS4-associated LS [50, 51].

Titrating doses of AAV-based gene therapy is also challenging. In the cardiac-specific *Taz* knock-out model of BTHS, higher doses of AAV9-CAG-hTAZ resulted in a higher percentage of transduced cells and greater improvements in the disease phenotype [25]. In a mouse model of FRDA, however, a dose of AAVrh10-CAG-hFXN that led to high-level expression of FXN caused cardiac toxicity [38]. Additionally, clinical studies in human patients have shown that high-dose AAV vector therapy could lead to serious adverse events and possibly death. It is crucial to provide a dose large enough to provide therapeutic efficacy but low enough to not cause toxicity. Adverse events seen in high dose AAV treatment have been associated with immunogenicity and liver toxicity [125, 126].

An immune response to an AAV vector-based therapy can be dangerous for the patient, and if directed against the AAV vector, usually in the form of neutralizing antibodies, it can also lead to reduced transgene expression and decreased therapeutic efficacy. Multiple strategies are being investigated to avoid these potential issues. Human clinical trials for AAV based gene therapy often include administration of corticosteroids to prevent or reduce immune responses [127]. Patients can be screened for the presence of neutralizing antibodies and treated with plasmapheresis prior to AAV administration [128]. New approaches, such as treatment with an endopeptidase that degrades IgG to lower the level of AAV neutralizing antibodies are also being explored [129].

In multi-system diseases, it can be challenging to decide which organ systems are most critical or if multisystem targeting is necessary for successful disease treatment. Strategies can include targeting the organ system that most impacts mortality or the system that most impacts quality of life. The studies discussed here also illustrates how preclinical studies can shed light on this problem. For example, data from treating the *Mck-*CKO model of FRDA indicate that targeting cardiomyopathy alone may not be sufficient to improve survival outcomes [36].

Testing new therapeutic options is challenging in mitochondrial diseases because of their rarity. For example, fewer than 100 cases of ethylmalonic encephalomyopathy have been identified worldwide [130]. Obtaining sufficient numbers of patients for a randomized controlled clinical trial of a gene therapy product for a mitochondrial disease will likely be impossible. Alternative trial designs with small numbers of patients or single patient “N of 1” trials will be necessary. The wide range of genetic and phenotypic variability displayed by mitochondrial diseases makes it challenging to develop broadly applicable interventions.

A yet unmentioned challenge of gene therapy is the significant cost. For example, Zolgensma, the AAV9 based therapy for spinal muscular atrophy, costs over $2 million. These high costs are due a variety of factors including development cost, high cost of manufacturing, limited numbers of companies developing therapies for ultra-rare diseases, and the small number of patients a specific therapy would likely benefit. A variety of strategies, innovative payment models, and policies are being explored with the goal of making these potentially life-saving therapies available to every patient in need [131].

### Future directions

Many laboratories are currently working on designing new capsids that exhibit enhanced transduction efficiency and higher specificity to target organs and cell types with the hypothesis that this will allow lower doses to achieve therapeutic efficacy. The mitochondrially targeted AAV developed by the Guy laboratory is particularly intriguing [118, 119]. Their vector is based on AAV2, but other mitochondrially-targeted serotypes could be developed using the same approach. This would enable capsids to target mitochondria within a specific cell type. Cell type-specific AAV capsid mutants would also reduce the risk of off-target effects potentially caused by serotypes with broader tropism. Cell type-specific AAV capsids could also reduce the need for alternative, potentially risky routes of administration, such as direct brain injection. This idea is well illustrated by AAV-PHP.B and AAV-PHP.eB.

AAV-PHP.B and AAV-PHP.eB are variants of AAV9 developed by targeted evolution that are much more efficient at transducing cells in the central nervous system than AAV9 [132]. These two serotypes were shown to be efficacious in the preclinical studies of *NDUFS4-*related LS [51] and *SLC25A46*-associated disease [108]. Unfortunately, AAV-PHP.B and AAV-PHP.eB do not show this enhanced brain transduction in primates [137]. This is due to the lack of a primate homolog for the AAV-PHP.B and AAV-PHP.eB transporter, lymphocyte activation protein 6a (Ly6a) [133–137]. Though these novel capsids are unlikely to be translated into human use, AAV-PHP.B and AAV-PHP.eB offer an excellent tool for preclinical murine studies, which have provided proof of concept that that capsid-engineered novel AAV vectors can dramatically improve transduction in the central nervous system [132]. Additional novel, highly brain penetrant AAV capsids continue to be developed and investigated [138]. These novel capsids could be critical for making gene therapy possible for many neurological diseases.

Gene therapies that could be applicable to multiple mitochondrial diseases would be incredibly advantageous. One potential target that may be applicable to multiple mitochondrial diseases is mitochondrial biogenesis. Pharmacologic mechanisms for accomplishing this in mitochondrial disease are discussed by Pitceathly et al*.* in a recent review [139]. The underlying hypothesis is that increasing mitochondrial biomass would lead to an overall increase in ATP production and restoration of redox balance. Systemic lentiviral delivery of *Peroxisome proliferator-activated receptor gamma coactivator 1-⍺* (*PGC-1⍺*), the master regulator of mitochondrial biogenesis, was neuroprotective in a mouse model of Alzheimer’s disease (AD) [140]. Mitochondrial dysfunction is increasingly recognized as a major contributor to the pathogenesis of AD, so this data suggests that *PGC-1⍺* gene therapy could have utility in other diseases involving mitochondrial dysfunction [140].

Attempts are also being made to pharmacologically target *NRF2* (*Nuclear factor erythroid 2-related factor 2*), another regulator of mitochondrial biogenesis. Mitochondrial myopathy patients receiving omaveloxolone, a potent NRF2 activator, for 12 weeks showed improvements in submaximal exercise tolerance and aerobic capacity, suggesting increased mitochondrial functions [141]. Patients with FRDA showed neurological improvement after 49 weeks of treatment with omaveloxolone [142]. This suggests that overexpression of *NRF2* by gene therapy could potentially be beneficial to patients with a variety of genetic mitochondrial diseases.

In addition to gene replacement therapy, there is increasing interest in gene editing based therapies using CRISPR and related technologies. CRISPR technology uses a guide-RNA (gRNA) to target a nuclease (most commonly Cas9) or base-editor to a very specific location in the genome. Depending on the nuclease used and the design of the gRNAs, insertions, deletions and base changes are possible. The components of a CRISPR-based therapy can be delivered using an AAV vector. The first CRISPR-based therapy tested in humans (EDIT-101) uses the AAV5 vector to deliver two specific gRNAs and *Staphylococcus aureus* Cas9 (SaCas9) (NCT03872479) [143]. SaCas9 is about 1 kilobase smaller than the commonly used *S. pyogenes* Cas9 (spCas9), allowing it to be combined with gRNA sequences in a single recombinant AAV vector genome [144]. EDIT-101 is for Leber congenital amaurosis (LCA) 10, a form of inherited retinal degeneration caused by a point mutation in the *CEP290* gene [145]. The intronic point mutation results in incorrect splicing and a truncated, non-functional CEP290 protein [145]. The pair of gRNAs and SaCa9 coded by EDIT-101 enable the excision of a short region containing the point mutation, resulting in correct splicing and production of functional CEP290 protein [145]. AAV-vector based CRISPR therapies are attractive because they enable gene therapy on genes larger than the AAV packaging capacity without needing to split the gene and deliver the pieces in multiple AAV particles. This strategy could be applicable to mitochondrial disorders caused by mutations in nuclear genes. However, like gene replacement therapy, it would require personalization in the form of specific gRNAs.

Delivery of gRNAs and the other CRISPR components to the mitochondria is challenging, limiting the technology’s utility in editing mitochondrial DNA. Recently, Hussain et al*.* have shown that the addition of a stem-loop element to gRNAs and a mitochondrial targeting sequence to Cas9 facilitates their import into the mitochondria [146]. Using their modified Cas9 and gRNAs the authors were able to show reduction of mtDNA with a specific mutation, indicating cleavage of mtDNA by Cas9 as linearized mtDNA is rapidly degraded [146]. Mok et al*.* developed a non-CRISPR-based mtDNA editing tool called DddA-derived cytosine base editor (DdCBE). DdCBE consists of a novel split bacterial cytidine deaminase (DddA), a mitochondrial targeting sequence, a TALE protein for specific DNA targeting and a uracil glycosylase inhibitor [147]. The authors were able to demonstrate highly specific editing of five mitochondrial genes [147]. This could be groundbreaking for treatment of mitochondrial diseases caused by mutations in the mtDNA. Both the modified CRISPR system and the DdCBE can delivered by AAV. Investigations into gene therapies targeting the mitochondrial genome are likely to be investigated.

## Conclusions

The preclinical studies presented here indicate that AAV vector-based gene therapy is a promising therapeutic modality in this disease space. The number of AAV vector-based gene therapy clinical trials initiated each year has been increasing since the early 2000s [148] and is likely to continue to increase throughout the next decade. As the numbers of trials and approvals continue to increase, we hope to see more gene therapy clinical trials for mitochondrial diseases in the near future and eventual approval of these products for use in these devastating diseases.

## Data Availability

Not applicable.

## Abbreviations

ETC: Electron transport chain
mtDNA: Mitochondrial DNA
MRT: Mitochondrial replacement therapy
AAV: Adeno-associated virus
ITR: Inverted terminal repeat
CNS: Central nervous system
BTHS: Barth syndrome
FRDA: Friedreich ataxia
LS: Leigh syndrome
EE: Ethylmalonic encephalomyopathy
MNGIE: Mitochondrial neurogastrointestinal encephalopathy
TK2: Thymidine kinase 2
LHON: Leber hereditary optic neuropathy
MLCL: Monolysocardiolipin
CL: Cardiolipin
FXN: Frataxin
Mck: Muscle creatine kinase
Pvalb: Parvalbumin
DRG: Dorsal root ganglia
Nse: Neuron-specific enolase
CB: CMV enhancer/β-actin
PDHC: Pyruvate dehydrogenase
CMV: Cytomegalovirus
IV: Intravenous
ICV: Intracerebral ventricular
MITRAC: Mitochondrial translational regulation assembly intermediate of cytochrome c oxidase
IT: Intrathecal
SDO: Mitochondrial sulfur dioxygenase
COX: Cytochrome c oxidase
SCAD: Short chain acyl-CoA dehydrogenase
TBG: Thyroxine-binding globulin gene enhancer
MDS: Mitochondrial DNA depletion syndrome
TP: Thymidine phosphorylase
HSC: Hematopoietic stem cell
UP: Uridine phosphorylase
dThd: Deoxythymidine
dAAT: α-1-Antitrypsin
HLP: Hybrid liver-specific
ALT: Alanine aminotransferase
AST: Aspartate transaminase
dC: Deoxycytidine
MFN2: Mitofusin 2
OPA1: OPA1 mitochondrial dynamin like GTPase
MICOS: Mitochondrial contact site and cristae organizing system
LCA: Leber congenital amaurosis
MT-ND4: Mitochondrial NADH dehydrogenase subunit 4
RGC: Retinal ganglion cell
COX8: Cytochrome c oxidase subunit 8
HSP: Mitochondrial heavy strand promoter
PGC-1⍺: Peroxisome proliferator-activated receptor gene coactivator 1-⍺
NRF2: Nuclear factor erythroid 2-realated factor 2
DdCBE: DddA-derived cytosine base editor
